# No Signs of Inclusive Fitness or Reciprocal Altruism in Advantageous Inequity Aversion

**DOI:** 10.1177/14747049231173401

**Published:** 2023-05-17

**Authors:** Jan Antfolk, Emmie Marklund, Irene Nylund, Annika Gunst

**Affiliations:** 1 Department of Psychology, Åbo Akademi University, Turku, Finland

**Keywords:** advantageous inequity aversion, inclusive fitness theory, reciprocal altruism, social development, adrenarche

## Abstract

Advantageous inequity aversion (i.e., the tendency to respond negatively to unfairness that benefits oneself) usually develops in 6–8-year-olds. However, little is known about the selection pressures that might have shaped this phenomenon. Using data collected from 120 4–8-year-old Finnish children, we tested two evolutionary explanations for the development of advantageous inequity aversion: reciprocal altruism (i.e., benefiting from sharing when the roles are likely reversed in the future) and inclusive fitness (i.e., benefiting from sharing with biological relatives that carry the same alleles). We first successfully replicated a previous experiment, showing that 6–8-year-olds display advantageous inequity aversion by preferring to throw away a resource rather than keep it for themselves. Here, this behavior was also displayed in 5-year-olds. Using a novel experiment, we then asked children to distribute five erasers between themselves, a sibling, a peer, and a stranger. That is, an equal distribution was only possible if throwing away one eraser. We found no support for advantageous inequity aversion being shaped by either inclusive fitness or reciprocal altruism. Future studies could investigate costly signaling and adherence to social norms to avoid negative consequences as ultimate explanations for advantageous inequity aversion.

Advantageous inequity aversion (AIA) describes the tendency to avoid and respond negatively to unfairness that would benefit oneself (Fehr & Schmidt, 1999). AIA has been shown to develop in 6–8-year-olds with some individual and cultural variation (e.g., Blake & McAuliffe, 2011; Fehr et al., 2008). The pattern does not differ between the sexes (e.g., Kogut, 2012; Qiu et al., 2017). The development of AIA seems to coincide with adrenarche (e.g., DePeretti & Forest, 1976; Kotler & Haig, 2018) and broader sociocognitive changes, such as theory of mind (e.g., Calero et al., 2013) and prosociality (e.g., Callaghan & Corbit, 2018; Knight & Kagan, 1977). The development of theory of mind has previously been associated with, for instance, fairer decisions in children (Tsoi & McAuliffe, 2020) and reciprocity to fair offers by other children (Schug et al., 2016; Takagishi et al., 2014). While some cultural variation exists, the available research suggests that inequity aversion is a cross-cultural phenomenon (Blake et al., 2015; Choshen-Hillel et al., 2020; Qiu et al., 2017; Shaw & Olson, 2012). Moreover, fairness norms can be found in most cultures in the world, including hunter-gatherer societies (Boehm, 2008; Gurven, 2004; Henrich, 2004). This raises the questions of whether and how AIA is shaped by natural selection. Despite the many advances in understanding the ontogeny of AIA, little is known about the selection pressures that might have formed this phenomenon, that is, why this age-dependent shift towards increased fairness has come to exist in the first place. Here, we designed a study to investigate the roles that inclusive fitness (Hamilton, 1964) and reciprocal altruism (Trivers, 1971) might have played in shaping AIA and its developmental timing. We also aimed to replicate an experiment from an influential study by Shaw and Olson (2012), in which AIA was found in 6–8-year-olds.

## The Developmental Timing of Advantageous Inequity Aversion

The developmental timing of AIA has been studied by having children of different ages choose between fair and unfair distributions of resources. For instance, in their seminal study, Fehr et al. (2008) investigated how 3–8-year-olds allocated candy between themselves and an absent partner. They found that most 3–4-year-olds favored themselves, whereas most 7–8-year-olds allocated candy, so neither they nor the partner benefited. Asking children to distribute erasers between two fictive children, Shaw and Olson (2012) found that 6–8-year-olds—despite generally disliking wasting resources—were more likely than 3–5-year-olds to throw away a resource instead of distributing resources unfairly. Studying how children distributed ten candies between themselves and another child, Kogut (2012) found that 5–6-year-olds tended to act in their own interest while 7–8-year-olds tended to behave more fairly. Similarly, Qiu et al. (2017) found that while 4- and 6-year-olds who took part in a spin-the-wheel lottery preferred a wheel where they had a higher chance of winning, 8-year-olds displayed AIA by preferring a fair spinning wheel. Together, these findings suggest that a developmental shift towards AIA occurs when children are around 6 years or slightly older.

The development of AIA occurs later than disadvantageous inequity aversion (DIA), that is, the tendency to reject and respond negatively to unfairness benefiting others over self. For instance, studying 4–8-year-olds, Blake and McAuliffe (2011) found that all age groups displayed DIA, while AIA could only be seen in older children. Similarly, Sheskin et al. (2014) found that DIA was displayed already in their youngest age group of 5–6-year-olds, whereas AIA was only present in the older age groups of 7–10-year-olds. In sum, with some variation within-age categories, there is support for the notion that older children display more AIA than younger children, whereas younger children already display DIA. This suggests that the two forms of inequity aversion develop independently and hints at separate evolutionary mechanisms behind AIA and DIA.

## Evolutionary Explanations of Advantageous Inequity Aversion

A possible ultimate explanation for AIA is reciprocal altruism (Trivers, 1971). Cooperative or helpful behaviors are favored by natural selection if they increase the fitness of the individual. Reciprocal altruism entails granting favors to others and thereby temporarily reducing one's own resources (at a relatively small risk of decreased fitness to self), with the expectation that the recipient will return the favor later (at a relatively large fitness benefit to self). Reciprocal altruism is especially likely when the fitness cost of the behavior is small, and the roles of the recipient and the distributor are likely to be reversed in the future. The evolutionary mechanism of reciprocal altruism necessitates a context in which individuals have sustained contact, such as within the family (in which case the mechanism can be parallel with that of inclusive fitness) or following the increased social interaction outside the family that coincides with adrenarche. Indeed, it has been demonstrated that reciprocity is more likely if there is a high probability for future interactions (Gächter & Falk, 2002). If AIA reflects reciprocal altruism, children are expected to be more prone to share resources with peers and close relatives, compared to strangers, as only the former are long-term relationships, which increases the chance of payback.

Another possible ultimate explanation of AIA is inclusive fitness. According to Hamilton's theory about inclusive fitness (1964), an individual benefits from sharing resources with an individual who likely shares the same allele copies because this increases the probability that these allele copies are propagated to future generations. An individual's fitness can improve if the cost to self of the altruistic act toward another individual is smaller than the benefit of the act to the recipient, after weighing the benefit by the relatedness between self and recipient. For example, Kotler and Haig (2018) argued that the tempo of human childhood development is largely shaped by its consequences on the fitness of close relatives. In line with this argumentation, the development of selfish behavior towards more fair behavior may be explained by individuals benefiting from sharing with close biological relatives. For instance, studies show that altruistic behavior in adults is positively associated with the degree of biological relatedness (e.g., Antfolk et al., 2017; Madsen et al., 2007). The theory of inclusive fitness would therefore predict that children are more prone to share with close relatives (e.g., a sibling) than with non-relatives (e.g., a peer or a stranger) irrespective of the likelihood of future interaction.

## Studying Evolutionary Explanations by Varying Partner Identity

One method for investigating ultimate explanations for AIA is to vary the partner's identity and observe how this modulates children's behavior. If the developmental shift towards fairness is an adaptation designed to promote reciprocal altruism, one would expect an increased AIA if the partner is a trusted long-term ally (e.g., a peer or sibling). If the developmental shift towards fairness originates in enhanced inclusive fitness, one would expect an increased AIA if the partner is a close relative (e.g., sibling, but not a peer). Previous studies have indeed varied the identity of the partner. For instance, by varying an absent partner's identity between someone from the same or another kindergarten/school, Fehr et al. (2008) demonstrated that the development of AIA coincided with an in-group preference and led them to propose that the same evolutionary mechanisms drive altruism and parochialism (i.e., a preference for favoring members of one's own social group). Similarly, Moore (2009) found that already 4–6-year-old children displayed more AIA if the partner was a peer than if the partner was a non-peer or a stranger. Although the results from these two studies align with the reciprocal altruism theory, neither study was designed to include also close relatives as partners and thereby test the inclusive fitness theory.

## The Current Study

In most of the previous studies on AIA, participants distributed resources to strangers (e.g., Fehr et al., 2008; Shaw & Olson, 2012). Testing evolutionary explanations necessitates a better understanding of how the relationship between the recipient and the child modulates AIA. Hence, we designed a study to test two possible explanations by observing how children simultaneously distribute resources between themselves and multiple recipients. The method was based on an influential study by Shaw and Olson (2012), in which the recipient was a fictive child, erasers were used as resources, and the participant had the option to throw away resources. In the current study, we designed a novel experiment in which participants distributed five erasers between themselves, a sibling, a peer, and a stranger. We examined to whom the participants distributed the erasers when it was impossible to distribute them equally without wasting any. We recruited 4–8-year-old children to participate in the study, as this age span should cover the developmental timing of AIA (e.g., Fehr et al., 2008; Kogut, 2012; Shaw & Olson, 2012).

Because our study drew on the setup used in Shaw and Olson (2012), our first goal was to replicate one (i.e., the fourth) experiment of their study, in which the authors demonstrated that 6–8-year-olds would rather throw away an extra resource than distribute it to themselves and create inequity. Even though the method developed by Shaw and Olson (2012) has been used in other studies, a replication of the fourth experiment has, to our knowledge, not been conducted. We extended the age span by including 4- and 5-year-olds. This enabled us to compare ages and to further explore at what age AIA develops. Based on the findings in the original study, we hypothesized the following:

- In the baseline task (where the resources can be distributed evenly), children in all age groups would prefer to distribute the resources rather than throw them away.
- In the experimental task, 4–5-year-olds would choose to distribute the resource at a frequency around chance level and thereby not display AIA.
- In the experimental task (where the resources cannot be distributed evenly), 6–8-year-olds would prefer to throw away the extra resource and thereby display AIA.

In the novel experiment, our second and main aim was to test two possible evolutionary explanations of AIA. We derived and tested the following hypotheses:

(iv) Based on the theory of reciprocal altruism, 6–8-year-olds were expected to distribute more erasers to themselves, their sibling, and their peer, than to the stranger.
(v) Based on the theory of inclusive fitness, 6–8-year-olds were expected to distribute more erasers to themselves and their sibling, than to their peer and the stranger.

## Materials and Methods

### Ethical Statement

The study was carried out in accordance with the Declaration of Helsinki. The Board for Research Ethics reviewed the research plan and granted ethical permission on October 23, 2019. Additionally, permission to recruit participants was given by the director of education and by the participating day-care centers, preschools, and primary schools. Before their child's participation, the legal guardians provided written informed consent. To ensure confidentiality, we assigned all children a number and noted the results from the experiments using this number. We kept the participants’ names and the corresponding numbers separate from the results.

### Participants

We recruited 4–8-year-old children from local day-care centers, preschools, and primary schools. In our first experiment, we included 24 children per age group, with a total sample of 120 children (51% girls). The children had to have a sibling to participate in our second experiment. For this reason, we excluded twelve children who participated in the first experiment from the second experiment. We also excluded two children that did not comprehend the instructions. In the second experiment, the sample thus consisted of 106 children (53% girls). Descriptive statistics are presented in Table 1.

**Table 1.** Description of the Participating Children.

Age groups	Experiment 1			Experiment 2		
	Boys (*n*)	Girls (*n*)	Mean age (SD)	Boys (*n*)	Girls (*n*)	Mean age (SD)
4-Year-olds	12	12	53.8 (3.4)	11	12	53.9 (3.5)
5-Year-olds	12	12	66.5 (3.7)	9	11	67.0 (3.7)
6-Year-olds	12	12	77.5 (3.6)	9	10	77.7 (3.5)
7-Year-olds^a^	11	13	88.1 (3.3)	10	13	88.1 (3.4)
8-Year-olds	12	12	101.1 (3.5)	11	10	101.2 (3.6)

*Note*. Mean age in months.

^a^ Due to recruitment error, there were more 7-year-old girls than 7-year-old boys.

The mean age of the total sample was 77.4 months (SD = 16.9, range 48–107) in the first experiment and 77.4 months (SD = 17.1, range 48–107) in the second experiment. Girls and boys did not differ in age (*t*[117.61] = 0.157, *p* = .875; *t*[101.26] = 0.13, *p* = .894, respectively).

### Materials

#### Background Information

The present study included a background form filled out by the child's legal guardian. We asked for the name, gender, and age of both the participant and the sibling closest in age to the participant. We also asked how the sibling and the participant are related (full-, half-, step-, or adoptive siblings). To measure how close the participant and the sibling are, we asked how long the siblings have known each other and how much time they spend together on a scale from 1 (*very little*) to 5 (*very much*).

#### Baseline Task

To measure how commonly children choose to throw away a resource, we used a baseline task identical to that in the first experiment by Shaw and Olson (2012), with the names adapted to Finnish/Swedish. In the task, we placed a paper with two squares in front of the participant. The squares contained letters representing two fictive children. We used star-shaped erasers with smiling faces as resources. We also placed a small trash can on the table should the participant choose to throw away any of the erasers. We put *four* erasers on the table and read the following:

“*Thank you for performing these tasks together with me. Earlier today, two kids named Benjamin and Oliver did a great job cleaning up their room, and we want to give them erasers as prizes. The problem is I don’t know how many erasers to give them. Can you help me with that? Great.*

*You get to decide how many erasers Benjamin and Oliver will get. We have these four erasers—one for Benjamin and one for Oliver.*

*Uh oh! We have two erasers left. Should I give one to Benjamin and one to Oliver, or should I throw them away?”*

We put the erasers in the squares while reading the script. We pointed towards the respective alternative (squares and trash can) when asking whether to distribute or throw away the eraser.

#### Experiment 1

The baseline task and the first experiment were separated by an unrelated survey task (Shaw et al., 2012) to obscure the aim of the study for the participants. Here, we read a brief scenario in which one child catches a fish, and another child takes the fish when the first child is not watching. The participant was then asked who owns the fish. In the first experiment, we replicated the fourth experiment in Shaw and Olson (2012), with the names adapted to Finnish/Swedish. Again, we used the paper with the two squares. Now, the squares contained letters representing the participant and the fictive child. We put *five* erasers on the table and read the following:

“*We want to give you some erasers for doing such a good job answering questions. We want to give some erasers to you and to another little boy (girl) named Emil (Emilia). The problem is I don’t know how many erasers to give to both of you. Can you help me with that? Great.*

*You get to decide how many erasers you and Emil (Emilia) will get. We have these five erasers. We have one for you, one for Emil (Emilia), one for you, and one for Emil (Emilia).*

*Uh oh! We have one left. Should I give this eraser to you, or should I throw it away?”*

Again, we put the erasers in the squares while reading the script and pointed towards the respective alternative (participant's own square and trash can) when asking whether to distribute or throw away the eraser.

#### Experiment 2

The first and second experiments were separated by a brief unrelated connect-the-dots task to give the children a moment's rest. In the second experiment, we used a paper onto which four squares had been drawn. Each square had a letter referring to the person the box belonged to. We again placed a small trash can on the table should the participant choose to throw away any of the erasers. The assistant then asked for the name of a buddy, explained that one of the squares belonged to that buddy, and told the child to whom the other squares belonged, that is, the child, the child's sibling, and a stranger called Alexander/Alexandra. To ensure the child understood the setup, the assistant asked the child to repeat to whom the squares belonged. This procedure was repeated if the participant failed. If the child spontaneously mentioned that the buddy was a relative, the assistant asked the child to name another buddy.

Next, the assistant took out *five* erasers and read the following:

“*Now you get to distribute some erasers between yourself, your sibling, your buddy, and Alexander (Alexandra) by putting the erasers in the squares. We have these five erasers. You get to distribute them as you like. You can also choose to throw away erasers; in that case, you throw them in the trash can. You can tell me when you are done.”*

Finally, the experimenter placed the erasers in front of the child, and they performed the task.

### Procedure

Before the actual data collection, we piloted the procedure with a few children to ensure that the instructions were comprehensible. The staff at the participating day-care centers, preschools, and primary schools aided us by providing all legal guardians with information about our study and inviting them to participate. As an incentive to participate, we gave each child a ticket to a local adventure park. The children could participate in the study when their legal guardians had filled in background information, signed, and returned the informed consent form.

The data collection occurred between November 2019 and February 2020 in a quiet space in the day-care centers, preschools, and primary schools. First, the assistant and the child sat down at a table. After this, the child partook in the task above. After the child had completed each task, the assistant told the child they had done a good job, and after completing the final task, the assistant asked them not to tell any of the other children about the tasks. Additionally, the assistant informed the child they would be awarded the erasers used in the task (but only after all participating children from the same day-care or school had completed their participation). Participation lasted approximately 15 minutes in total.

### Statistical Analyses

We conducted all analyses using the platform *R* (R Core Team, 2021). In the baseline task and the first experimental task, we analyzed data using binomial tests to examine whether the participants preferred to distribute or throw the extra resource(s). A sample size of 42 is needed for 80% power (1 − *β*) probability of observing a statistically significant (*α* = .5) result if the null hypothesis proportion is 50% and the expected true proportion is 70%. Additionally, we used chi-square tests to determine whether the probability of sharing or throwing erasers differed as a function of age and gender. A Chi-squared test with 4 degrees of freedom, a sample size of 75 is needed to have 80% power to observe an effect of *w* = .4. For 1 degree of freedom, a sample size of 50 is needed. In the second experiment, we first analyzed the sharing behavior by conducting an ANOVA for each recipient option (*oneself*, *sibling*, *peer*, *stranger*, or *throw away*) with age as the independent variable. For a between-subjects ANOVA with five groups, a sample size of 200 is required to reach 80% power to observe an effect of *f* = .25. Next, we conducted one ANOVA per age group to analyze the within-age pattern of distributed erasers to the recipient options. For a within-subjects ANOVA with five levels, a sample size of 22 is needed to have 80% power to observe an effect of *f* = .25, assuming a within-subjects correlation of *r* = .05. Tukey post hoc tests (*α* = .05) were used to follow up on the results. To analyze inequity aversion more specifically, we analyzed the number of even and uneven distributions of erasers. A distribution with one eraser distributed to each recipient and one thrown away was considered even, and any other uneven. A Chi-squared test was then performed to analyze the association between age and type of distribution. Binomial tests were used to analyze the proportion of even and uneven distributions within age groups.

## Results

### Replication

For the baseline task, a binomial test showed that the children were more likely to share the extra resource than to throw it away (95 out of 120, 79.2% [70.80%, 86.04%], *p* < .001). This demonstrated that children are unwilling to throw away resources when inequity is not involved, supporting our first hypothesis. There were no significant differences due to age (*χ*^2^[4] = 7.58, *p* = .108) or gender (*χ*^2^[1] = 0.30, *p* = .587) regarding whether the children shared or threw away the extra resource.

**Figure 1.**
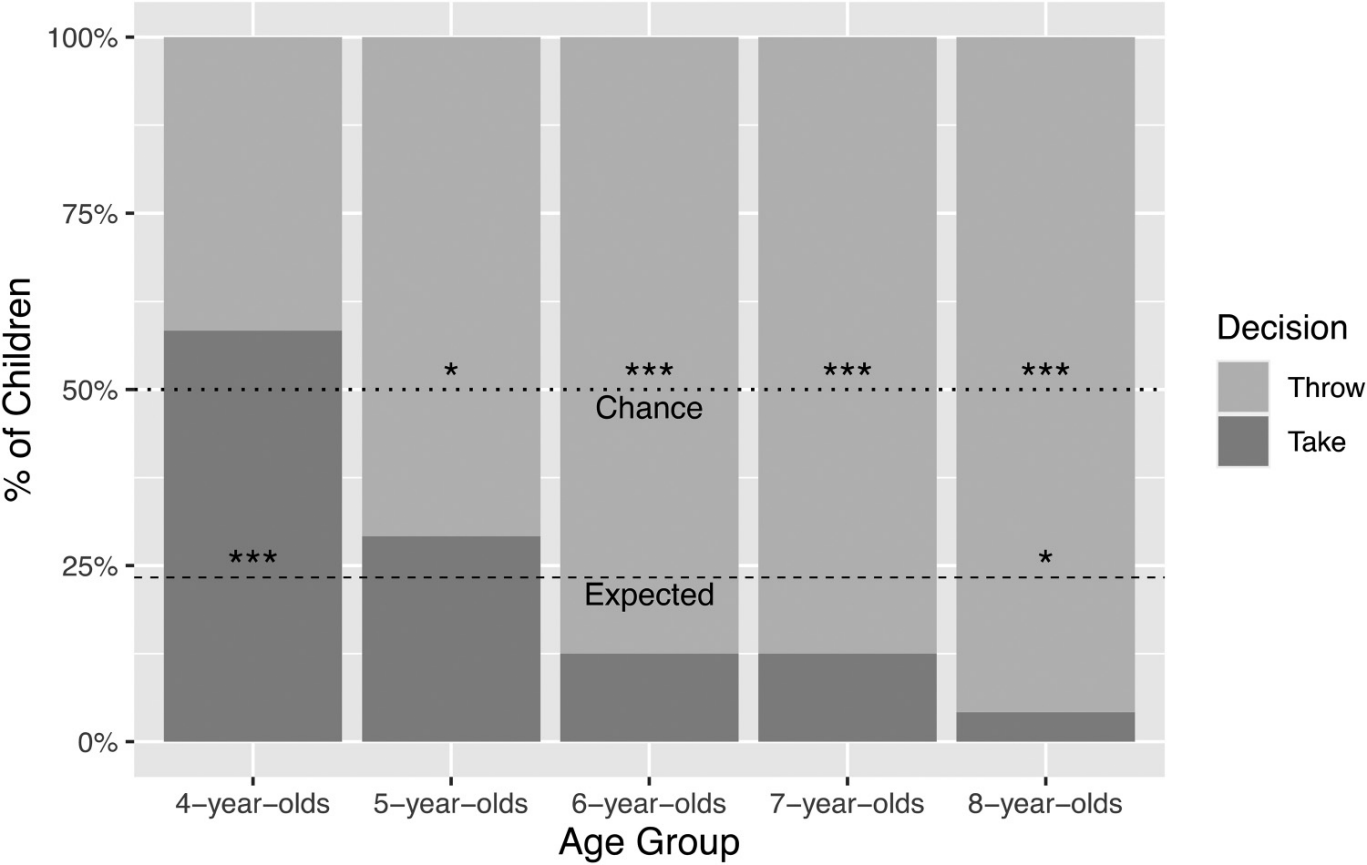
Advantageous inequity aversion by age group.

*Note.* Decisions to throw away (lighter gray) or take (darker gray) a resource by age group (*x*-axis). The *y*-axis represents the percentage of children in a particular age group making a specific choice. The 50% chance level (i.e., children are randomly taking or throwing resources) is marked with a dotted line. Statistically significant deviation from the chance value is marked with asterisks and signifies advantageous inequity aversion. The expected level of 23.33% (i.e., the projected rate if age and decision are not associated) is marked with a dashed line. Statistically significant deviations from this level are also marked with asterisks. ***p < .001. *p < .05.

Decisions to throw away or take a resource in the first experiment are depicted in Figure 1. A binomial test showed that the children were less likely to take the extra resource than to throw it away (28 out of 120, 23.33% [16.10%, 31.28%], *p* < .001). This demonstrated an overall preference for throwing away the resource to avoid advantageous inequity. As expected, there were significant age differences in the experimental task (*χ*^2^[4] = 24.97, *p* < .001). A post hoc test showed that the observed decisions of the 4-year-olds were above (*p* < .001) and that the observed decisions of the 8-year-olds were below (*p* = .013) the expected value (i.e., 23.33%).

Whereas the other age groups were more likely than not to throw away the resource, 58% of the 4-year-olds chose to take the resource themselves, partly supporting our second hypothesis (that 4–5-year-olds would distribute the resource at chance level) and fully supporting our third hypothesis (that 6–8-year-olds would prefer to throw away the extra resource). Only 4% of the 8-year-olds decided to take the resource themselves. There was no significant gender difference (*χ*^2^[1] = 0.30, *p* = .585).

### Evolutionary Explanations

**Figure 2.**
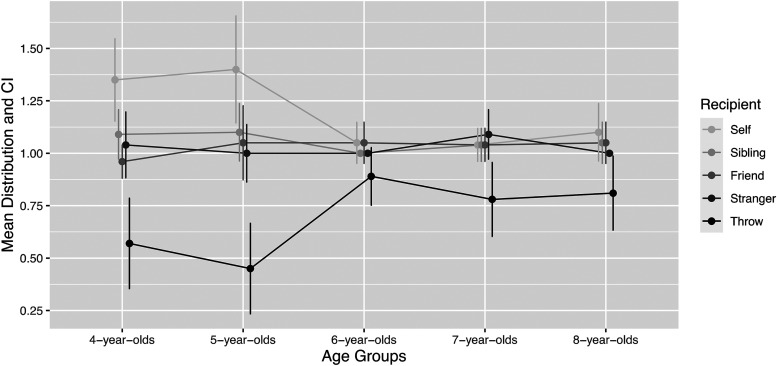
Resource distribution by recipient type and age group.

*Note*. Resource distribution by recipient type (lines) and age groups (*x*-axis). The mean distributions of the five resources are plotted on the *y*-axis and error bars represent the 95% confidence intervals (CI).

Table 2 shows the mean number and range of resources distributed to the different recipients in the second experiment. The distribution indicated an overall self-preference, as the children took most of the resources themselves and tended to avoid throwing away resources. There were no statistically significant differences in resources distributed to the sibling, the peer, and the stranger (see confidence intervals in Figure 2), providing evidence against both the reciprocal altruism hypothesis and the inclusive fitness hypothesis.

**Table 2.** Number of Erasers Distributed to Possible Recipients.

Recipient	Mean (SD)	Range
Oneself	1.19 (0.42)	1–3
Sibling	1.06 (0.23)	1–2
Peer	1.03 (0.26)	0–2
Stranger	1.03 (0.26)	0–2
Throw away	0.70 (0.46)	0–1

*Note.* 0–5 is the maximum range of resources that could be distributed across all five possible recipients.

We first analyzed how the children's age was associated with how many resources were given to each of the five possible recipient options. We performed one ANOVA test for each recipient option. There was an association between age and the amount of resources children took for themselves (*F*[4] = 4.01, *p* = .005, Figure 2). The 5-year-olds (*M* = 1.40, SD = 0.60) took more erasers for themselves compared to 6-year-olds (*M* = 1.05, SD = 0.23), but the post hoc Tukey test was not statistically significant (*p* = .054). The difference between 5-year-olds and 7-year-olds (*M* = 1.04, SD = 0.21) was, however, statistically significant (*p* = .031).

There was also a significant association between age and the number of erasers thrown away (*F*[4] = 3.61, *p* = .009). A post hoc test showed that 6-year-olds (*M* = 0.89, SD = 0.32) threw away more erasers than did 5-year-olds (*M* = 0.45, SD = 0.51, *p* = .018) and 4-year-olds (*M* = 0.57, SD = 0.51), although the latter difference was not statistically significant (*p* = .119).

There were no statistically significant effects of age on the number of erasers distributed to a sibling, a peer, or a stranger. The results indicated a self-preference among the younger children, which disappeared with age, with a significant shift in the age between 5 and 7. Rather than this shift reflecting an increased preference for a sibling, peer, or stranger, this shift reflected an increased will to throw away the extra resource.

### Inequity Aversion and Fairness

Because inequity aversion would be expressed as a perfectly even distribution of erasers, with one eraser given to each recipient and one eraser thrown away, we further analyzed the pattern of distributed erasers according to age group (Table 3). There were notably higher rates of even distributions in 6–8-year-olds compared to 4–5-year-olds. A chi-square test showed a significant association between age and type of distribution of erasers (*χ*^2^[4, 106] = 13.27, *p* = .010).

**Table 3.** Frequency of Type of Distribution by Age Group.

	4-Year-olds	5-Year-olds	6-Year-olds	7-Year-olds	8-Year-olds
Even	13	9	17	18	17
Uneven	10	11	2	5	4

*Note.* A distribution with one eraser distributed to each recipient and one thrown away was labeled an even distribution. Any other pattern of distribution was labeled an uneven distribution.

To test whether distributions differed from chance level (50%) in each age group, we analyzed the data using binomial tests. The binomial tests showed that 4- and 5-year-olds distributed the erasers evenly or unevenly at chance levels (*p* = .678 and *p* = .824), and 6-year-olds (*p* = .001), 7-year-olds (*p* = .011), and 8-year-olds (*p* = .007) distributed the erasers significantly more often evenly than unevenly. These results further support the notion that 6–8-year-olds are more averse to inequity than 4–5-year-olds and that this aversion, expressed as a significant majority of the distributions being even, can be seen already in 6-year-olds.

## Discussion

Using data collected from 4- to 8-year-old children in day-care centers, preschools, and schools in Finland, we aimed to test two different evolutionary explanations for the development of AIA: reciprocal altruism and inclusive fitness. Our findings did not provide any support for either hypothesis. We also replicated an experiment by Shaw and Olson (2012), in which 6–8-year-olds displayed AIA.

### Replication of Experiment With Option to Throw Away Resource

In the baseline task, we found that children in all age groups preferred to distribute the two extra resources when neither of the options to distribute or throw away the resource resulted in inequity. This was in line with the original study by Shaw and Olson (2012), who found similar behavior for their sample of 6–8-year-olds. Our findings thus strengthen the support for the idea that if children throw away resources to avoid inequity, such behavior can be considered a display of inequity aversion.

In the experimental task, we hypothesized that the 6–8-year-olds would more likely throw away the resource compared to the 4–5-years-olds since research has shown that AIA tends to develop around the age of 6 or later (Fehr et al., 2008; Shaw et al., 2016). However, we found that children overall preferred to maintain equity instead of distributing the extra eraser to themselves. Surprisingly, this was the pattern in all age groups except for the youngest (i.e., the 4-year-olds)—that is, also the 5-year-olds displayed AIA. Because the sample in the original study (Shaw & Olson, 2012) only included 6–8-year-olds, the hypothesis about the younger participants’ behavior was not based on the original study. However, even though research suggests that AIA develops after around 6 years (Fehr et al., 2008; Shaw et al., 2016), it is not a sharp shift but a gradual shift that takes place in different children at slightly different ages. Given that Finland is a Western country with a relatively high focus on equality, the timing of the shift could, to some extent, also be explained by local factors related to cultural norms and wealth. Worth noting is also that in the second experiment, including a sibling, a peer, and a stranger, both the 4-year-olds and the 5-year-olds created unequal distributions more often than the older children, demonstrating a self-preference. Although the younger children took more for themselves compared to the older children, the only statistically significant difference was between 5- and 7-year-olds. As this statistical test was somewhat underpowered, the non-significant differences might also be due to the relatively small sample size. Moreover, the behavior of the 4-year-olds resembled the behavior of the older children to a greater extent than expected. This might be explained by the fact that the experimental task was complex and hence might have been too difficult for the youngest age group to fully understand, leading to increased randomness in their responses.

### Evolutionary Explanations of Advantageous Inequity Aversion

The novel experiment did not yield any support for the hypotheses that inclusive fitness or reciprocal altruism underlies the development of AIA: There were no age-related patterns of increased distribution of resources to a sibling, a peer, or a stranger. As the developmental timing of AIA coincides with adrenarche, our results do not provide any support for the social and cognitive shifts that coincide with adrenarche have been shaped by inclusive fitness or reciprocal altruism (Weisner, 1996).

One alternative ultimate explanation for AIA is costly signaling. According to the theory of costly signaling, individuals signal favorable traits with altruistic acts (e.g., sharing resources without expecting anything in return) to convey one's desirability as a mate or ally, thus indirectly increasing one's fitness (Gintis et al., 2001). Another related ultimate explanation for AIA is adherence to social norms regarding fairness and striving to maintain one's reputation to avoid punishment (Fehr & Fischbacher, 2003). Our results align with these alternative hypotheses: Sharing more equally was not due to some other recipient becoming more important but rather more general unselfishness (i.e., oneself becoming equally important as all others). Previous results by Shaw et al. (2014) also demonstrated that children do not act as fair when they think no one knows about their decision. Corroborating this, some of the children in our study stated that they would take more for themselves if no one were watching. It is, therefore, possible that inequity aversion stems from children selectively abiding by norms of fairness; only when being observed.

Our results are also in line with Smith et al. (2013), who found that only 7–8-year-olds shared equally when given the option to keep or share stickers with another child, even though younger children also seemed to be aware of norms of fairness. This indicates that being aware of norms of fairness is insufficient for explaining AIA, as both younger and older children understand the norm, but only older children adopt it. Some have suggested that the changes occurring during childhood are more about a growing preference to signal impartiality and appear fair to others (Shaw & Olson, 2014; Shaw et al., 2014, 2016). The puzzling question that remains is why children develop a preference for fairness or at least for appearing fair, expressed as inequity aversion, at around age 6.

### Individual Differences in Developmental Timing

At group level, we found that older children displayed more AIA. However, all the older children did not show AIA by distributing the erasers evenly. For example, some of the participating children in our study seemed to think about their decisions for a longer time than others, and some children distributed the erasers and then changed their minds. This might reflect individual differences in neurophysiological maturation (e.g., adrenarche) or experience, leading some children to reflect upon and, in some cases, alter their decision. The participants also seemed to adopt different strategies. Some children verbally stated that one should be fair, while others said one should not waste. In sum, different strategies and personal attributes, possibly influenced by values and socialization by parents and peers, may play a part in individual sharing behavior. In this way, culture may have an impact on inequity aversion.

### Limitations and Future Directions

Some limitations need to be considered when interpreting the results. First, relatively low statistical power in the between-subjects ANOVA test may account for the fact that we did not obtain statistically significant differences between all younger and all older age groups. To rule out type-II errors, future studies could replicate this analysis in larger data.

The tasks in the present study do not fully correspond to situations that children normally face in resource-distributing contexts and might therefore have been slightly unrealistic. One could speculate that the environment, a school setting with standard principles of behavior and value systems, could affect how fair the children acted. When the participants were introduced to the study, the word “tasks” was used. This, in combination with the school environment, might have implied that there is a right and wrong answer to the tasks. This could have influenced the participants to distribute the erasers in a manner they presumed to be expected. It is also possible that our non-significant results for the reciprocal altruism and inclusive fitness hypotheses were due to the children distributing resources to different partners simultaneously. For comparison, in the study by Fehr et al. (2008), in which the authors found an in-group preference, the children did not distribute resources to the in-group member and the out-group member simultaneously. Moreover, participants were told “good job” after each task, and this feedback may have affected the results of our study by reinforcing individual responses. These issues could be addressed in future research by clarifying that there is no right or wrong answer. It is also possible that some children, although encouraged not to, told each other about the tasks, which could impact their choices.

After completing the tasks, some participants said they did not understand that they would receive the erasers. This may have affected the way the participants distributed the erasers. Similarly, some participants did not seem to understand that the choice to throw away any eraser led to them actually being thrown away. One participant wanted the experiment leader to confirm that the trash can was not real and that the eraser would be picked up afterward. Another participant even politely picked up the thrown eraser after the study ended and gave it back to the experiment leader. Future research could address these limitations by ensuring participants understand that they receive the erasers distributed to themselves and that the erasers are actually thrown away.

The experiment leaders had expectations of the participants’ behaviors based on the hypotheses. These expectations might have affected, for example, how the options in the tasks were emphasized and could thereby have influenced the participants’ decisions. The experiments were run in a fixed order and not counterbalanced. Moreover, in the experiment exploring possible ultimate explanations for AIA, the children had the option to distribute the erasers evenly by giving each person one eraser and throwing away the last one. As distributing the last eraser to any other person than themselves would have created a situation of disadvantageous inequity (i.e., another person receiving more erasers than oneself), it could be that DIA overrode any preference for distributing the last eraser to the peer, sibling, or stranger. Future studies could rectify this limitation by using the same procedure as in experiment 1 and varying the identity of the partner. If, for example, the hypothesis of inclusive fitness holds true, the children should be more likely to throw away the resource when the partner is a stranger compared to when the partner is a sibling.

Lastly, there are several extraneous variables that might moderate the observed behavior, including the quality of the relationship between the known recipients, prior experiences related to reciprocity, as well as the gender of the sibling and the friend. For a more detailed account of how such variables might affect decisions in this setting, more research is warranted. We also did not assess the socioeconomic status of the children. It could be that the children's decisions are affected by how valuable they consider the erasers. This concerns most studies in the field that have used similar designs. However, Finland is a country with a small wealth gap in global comparison (World Population Review, 2023). It is likely that most children in our study considered the value of the resource as low, as Finnish schools usually provide the pupils with materials (including erasers). Therefore, we do not expect socioeconomic status to play a major role in our study.

## Conclusions

We first successfully replicated an experiment by Shaw and Olson (2012), showing that 6–8-year-olds display AIA by preferring to throw away a resource rather than keeping it for themselves. Here, this behavior was also displayed in 5-year-olds. Our novel experiment did not yield any support for the hypotheses that inclusive fitness or reciprocal altruism underlies the development of inequity aversion. Future studies could investigate costly signaling and adherence to a fairness norm to avoid punishments as ultimate explanations for AIA.
